# Clinical Safety and Efficacy of Pegcetacoplan in a Phase 2 Study of Patients with C3 Glomerulopathy and Other Complement-Mediated Glomerular Diseases

**DOI:** 10.1016/j.ekir.2023.08.033

**Published:** 2023-08-25

**Authors:** Bradley P. Dixon, Larry A. Greenbaum, Liwei Huang, Sandeep Rajan, Chunlei Ke, Yiwei Zhang, Li Li

**Affiliations:** 1 Renal Section, Department of Pediatrics, University of Colorado School of Medicine, Aurora, Colorado, USA; 2 Emory University and Children’s Healthcare of Atlanta, Atlanta, Georgia, USA; 3 Tidewater Kidney Specialists, Inc, Chesapeake, Virginia, USA; 4 Vanderbilt University Medical Center, Nashville, Tennessee, USA; 5 Apellis Pharmaceuticals, Inc., Waltham, Massachusetts, USA

**Keywords:** complement, C3 glomerulopathy, end-stage kidney disease, glomerulonephritis, pegcetacoplan, proteinuria

## Abstract

**Introduction:**

Dysregulated complement activation is likely the primary driver of disease in C3 glomerulopathy (C3G) and contributes to other complement-mediated diseases, including immunoglobulin A nephropathy (IgAN), lupus nephritis (LN), and primary membranous nephropathy (PMN). No complement inhibitors are proven to halt disease progression in these diseases. Pegcetacoplan, a targeted C3 and C3b inhibitor, may mitigate complement-mediated kidney damage in C3G and other glomerular diseases in which complement may have a pathogenic role.

**Methods:**

This open-label, phase 2, 48-week study evaluated the preliminary efficacy and safety of subcutaneous pegcetacoplan for patients with complement-mediated glomerular diseases. The primary end point was proteinuria reduction, measured as 24-hour urine protein-to-creatinine ratio. Secondary end points included remission status, changes in estimated glomerular filtration rate (eGFR), and pharmacodynamic biomarkers. Treatment-emergent adverse events (TEAEs) were monitored.

**Results:**

Efficacy results for the C3G cohort are reported herein, along with safety results for the study population. In the C3G cohort, mean proteinuria reduction from baseline to week 48 was 50.9% in the intent-to-treat (ITT) population (*n =* 7) and 65.4% in the per-protocol (PP) population (*n =* 4). Mean serum albumin normalized and mean eGFR was stable over 48 weeks. Mean serum C3 levels increased 6-fold and mean soluble C5b-9 levels decreased by 57.3% at week 48. The most common adverse events (AEs) were upper respiratory tract infection, injection site erythema, nausea, and headache. No meningitis or sepsis cases were reported, and no serious treatment-related AEs were observed.

**Conclusion:**

Pegcetacoplan may provide therapeutic benefit for C3G and has a favorable safety profile across the 4 glomerular diseases studied.

## Graphical abstract

**Figure:**
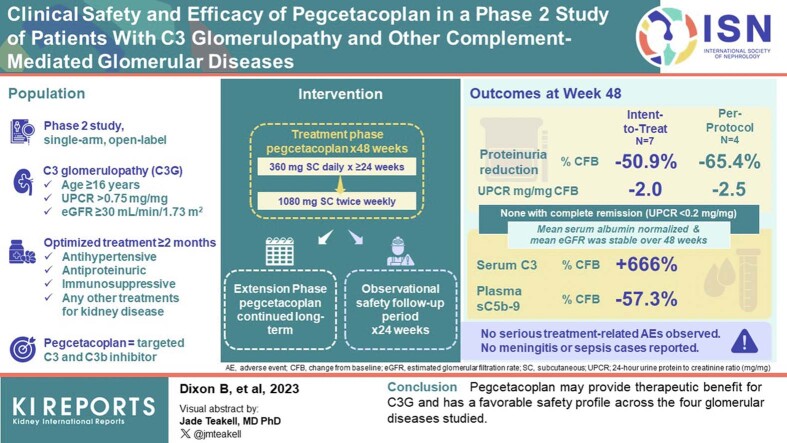

The complement system contributes to innate and acquired immunity through activation of the classical, lectin, and alternative pathways.^1^^,^^2^ Activation of the complement pathways triggers a cascade, leading to inflammation, as well as opsonization and subsequent phagocytosis and cellular lysis, and ultimately elimination of an invading pathogen or damaged tissue.^3^^,^^4^ These 3 activation pathways converge at C3, which plays a central role in the complement system and is key for activation of downstream terminal pathways, including formation of the membrane attack complex.^5^^,^^6^

Dysregulated activation of complement has been implicated in the pathogenesis of various forms of glomerulonephritis, including C3G, IgAN, LN, and PMN.^7^^,^^8^ The etiology of complement dysregulation in C3G may be genetic susceptibility (e.g., susceptible haplotypes or pathogenic mutations), autoimmunity such as autoantibodies against complement regulatory factors, or other environmental factors.^1^, ^2^, ^3^, ^4^, ^5^, ^6^, ^7^^,^^9^, ^10^, ^11^ Dysregulation of the complement system in these glomerular diseases, especially in C3G, results in amplification and propagation of the complement cascade, including downstream membrane attack complex (C5b-9) assembly (Figure 1), culminating in glomerular deposition of C3 and C5 breakdown products and kidney injury.^4^^,^^12^

**Figure 1:**
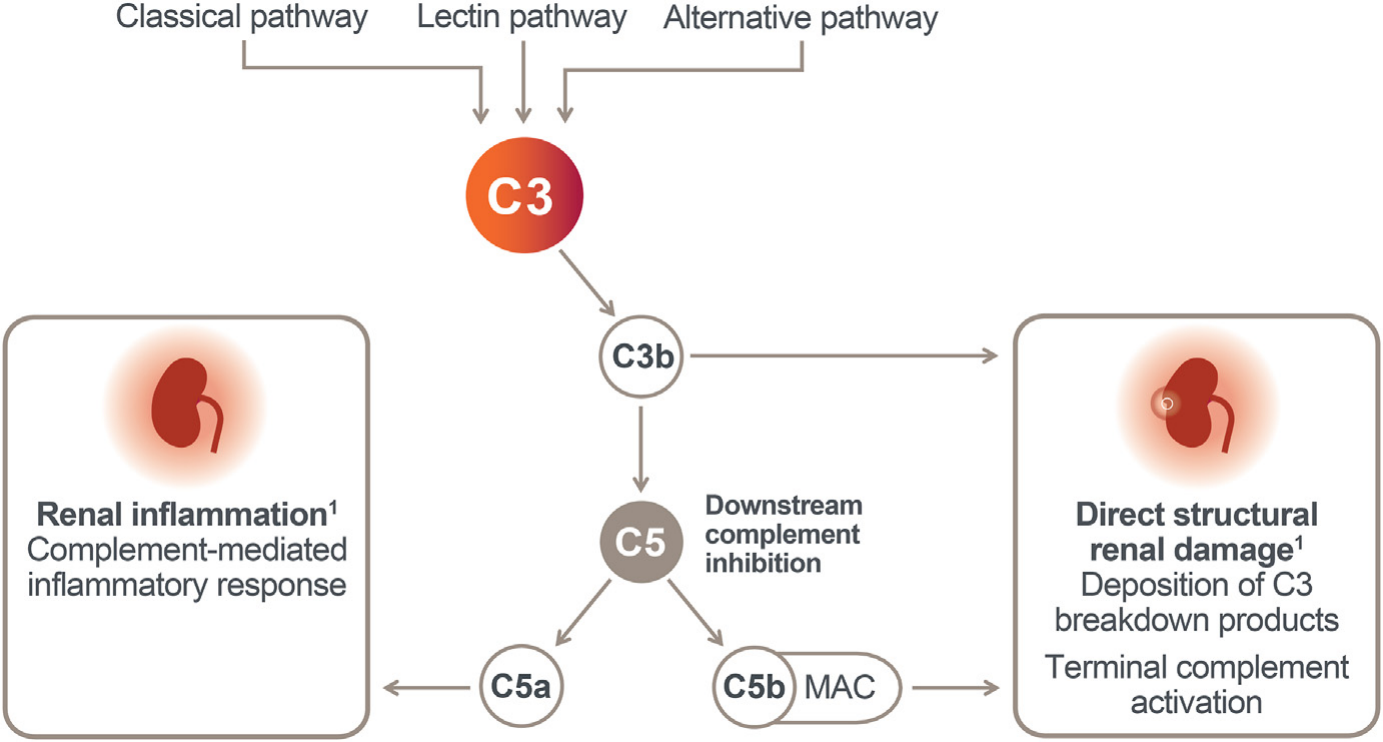
Role of the complement pathway in glomerulonephritis. Complement activation with or without immunoglobulin deposition is achieved through the classical, alternative, and/or lectin complement pathways, which converge to form a C3 convertase that converts C3 to its active split product C3b. Upon C3 cleavage, the amplification loop promotes rapid generation of more C3 convertase (C3bBb). Released C3b mediates opsonization for phagocytosis, leading to deposition of C3 breakdown products in glomeruli. Activation of C3b through factor B and factor D also results in formation of a C5 convertase complex that activates the terminal effector pathway and cleaves C5 into C5a and C5b. Whereas C3a and C5a trigger inflammatory response in tissue, C5b binds with C6, C7, C8, and C9 to form the MAC, which causes cell lysis. MAC, membrane attack complex.

C3G is a rare disease, with an incidence of 1 to 2 cases per million people.^13^ It often presents during adolescence, though sometimes is not diagnosed until adulthood.^13^, ^14^, ^15^ Dysregulation of complement, leading to deposition of C3 and C5 breakdown products in the kidney, is the primary driver of C3G disease.^4^^,^^6^^,^^13^^,^^14^ The prognosis of C3G is often poor, with 30% to 50% of patients progressing to end-stage kidney disease within 10 years of diagnosis.^16^, ^17^, ^18^ Efforts are ongoing to characterize disease features that predict prognosis and to determine early clinical end points that can predict long-term kidney function.^16^, ^17^, ^18^ Although kidney transplantation is an option, disease recurrence is common, with allograft loss due to recurrence in up to 50% of transplanted patients.^10^

Pegcetacoplan, a targeted C3 and C3b inhibitor, is in development for the treatment of C3G, where it has the potential to address the primary pathogenic driver of disease.^19^ By inhibiting C3 and C3b, pegcetacoplan may prevent excessive glomerular deposition of C3 and C5 breakdown products and the ensuing glomerular inflammation and renal damage observed in this disease (Figure 1), which in turn may translate to improved clinical outcomes.^19^, ^20^, ^21^ Therefore, the objective of this phase 2 DISCOVERY study was to investigate the preliminary efficacy and safety of pegcetacoplan over 48 weeks as a potential treatment for patients with the complement-mediated glomerular diseases of C3G, IgAN, LN, and PMN. The low enrollment and high rate of discontinuation limited assessment of the IgAN, LN, and PMN cohorts. Efficacy results for the C3G cohort are reported herein, along with safety results for the entire study population.

## Methods

### Study Design

This single-arm, open-label, phase 2 study (NCT03453619) evaluated preliminary efficacy and safety outcomes with the use of pegcetacoplan in patients with complement-mediated glomerulopathies over a 48-week treatment period. The trial was conducted in accordance with Good Clinical Practice guidelines and the principles of the Declaration of Helsinki. The protocol and associated documents were approved by the institutional review board or independent ethics committee before initiation at each site.

**Figure 2:**
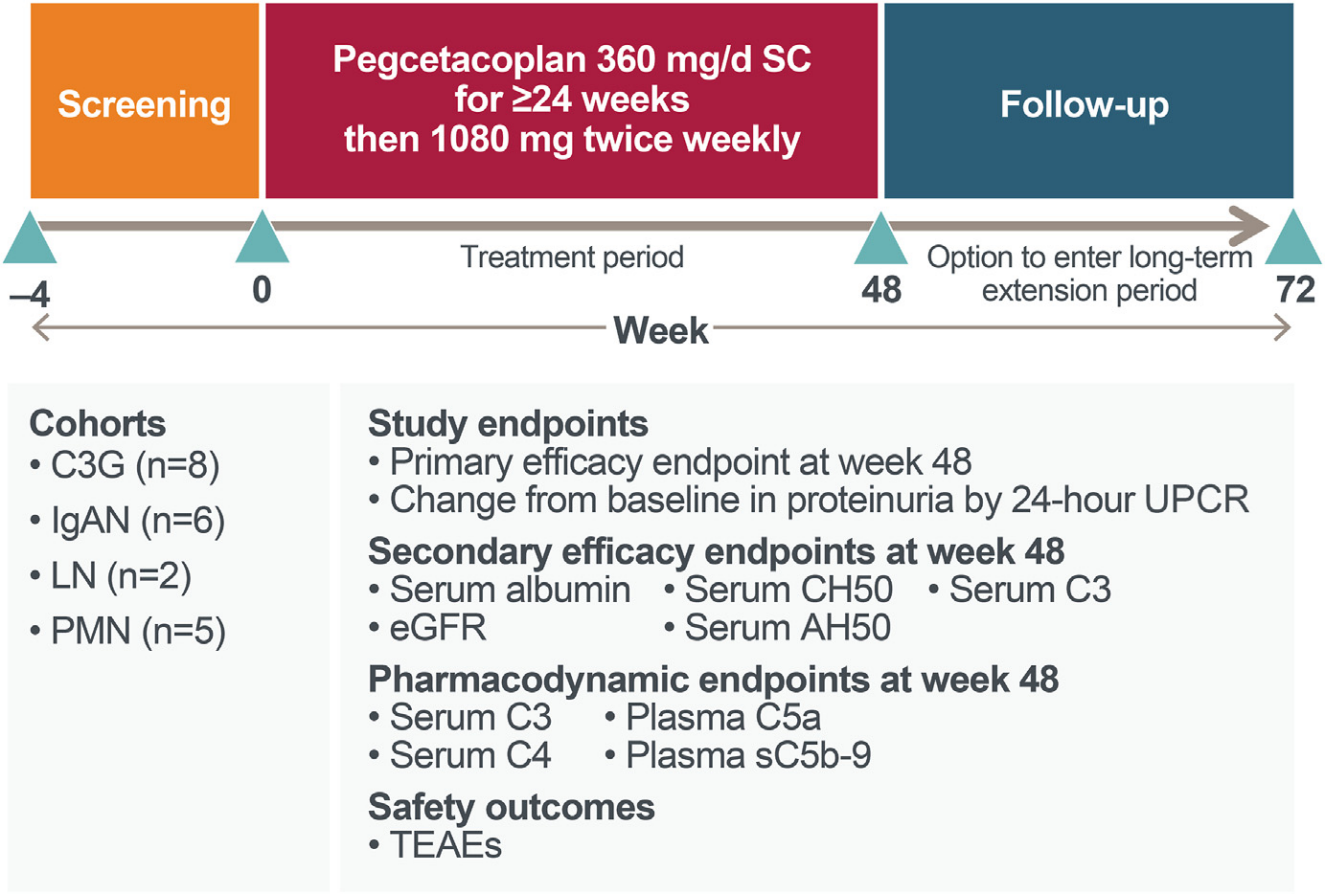
Study schema. This phase 2 open-label study included a screening period, a 48-week treatment period, 24-week observational safety follow-up, and an optional long-term extension phase. Starting on day 1, 4 patient cohorts received self-administered daily SC doses of 360 mg of pegcetacoplan until at least week 24 and then could be switched over to twice weekly dosing with 1080 mg as early as week 24. Efficacy and safety end points were assessed at baseline and at the end of the treatment period at week 48. AH50, alternative complement pathway hemolytic activity; C3G, C3 glomerulopathy; CH50, classical pathway hemolytic activity; eGFR, estimated glomerular filtration rate; IgAN, immunoglobulin A nephropathy; LN, lupus nephritis; PMN, primary membranous nephropathy; SC, subcutaneous; sC5b-9, soluble C5b-9; TEAE, treatment-emergent adverse event; UPCR, urine protein-to-creatinine ratio.

Pegcetacoplan was administered as a 360 mg subcutaneous infusion daily through at least week 24, after which patients could transition to a dosing regimen of 1080 mg twice weekly. After completion of the 48-week treatment period, patients who in the opinion of the investigator were experiencing clinical benefit from pegcetacoplan were invited to participate in a long-term extension phase to continue to receive treatment with pegcetacoplan until commercially available for the disease under treatment. Those who did not participate in the long-term extension phase were expected to complete a 24-week observational safety follow-up (Figure 2). Data reported herein are for the first 48 weeks of treatment. Patients were expected to be on a stable and optimized treatment regimen for their disease, in the opinion of the investigator, for at least 2 months prior to the first dose of pegcetacoplan; therapies included, but were not limited to, antihypertensive, antiproteinuric, and immunosuppressive agents.

### Enrollment Criteria

Key inclusion criteria for the C3G cohort were age ≥16 years at screening; diagnosis of C3G confirmed by low C3 level or historical kidney biopsy; presence of proteinuria, defined as a 24-hour urine protein-to-creatinine ratio (UPCR) >750 mg/g; eGFR ≥30 ml/min per 1.73 m^2^; and stable or worsening kidney disease while on stable and optimized treatment for ≥2 months before the first dose of pegcetacoplan. In addition, patients were required to receive vaccinations against encapsulated bacteria, including *Neisseria meningitidis*, *Streptococcus pneumoniae*, and *Haemophilus influenzae* type B at least 2 weeks prior to the first dose of pegcetacoplan. The study aimed to exclude patients with kidney disease secondary to another condition (e.g., infection, malignancy, monoclonal gammopathy, and medication). Exclusion criteria, as well as inclusion criteria for patients with IgAN, LN, and PMN, are listed in Supplementary Table S1.

### Outcomes

The primary efficacy end point was change in proteinuria from baseline (CFB; the first day of study drug administration) to week 48. Proteinuria was measured by 24-hour UPCR. Secondary efficacy and pharmacodynamic end points included complete clinical remission, defined as normalization of proteinuria (i.e., UPCR of <200 mg/g at week 48); stabilization or improvement in eGFR from baseline to week 48 (eGFR at week 48 no more than 25% less than the baseline); and changes in serum and plasma disease biomarkers (albumin, C3, C4, C5a, soluble C5b-9 (sC5b-9), classical pathway hemolytic activity [CH50], and alternative complement pathway hemolytic activity [AH50]).

### Statistical Analysis

Given the exploratory nature of the study, no formal statistical hypothesis testing was performed, and thus sample sizes were not based on statistical power of the study. The ITT population included all patients who received ≥1 dose of pegcetacoplan. The primary efficacy end point analysis was based on patients in the ITT population who had both baseline and week 48 data (*n* = 7). The PP population included all patients in the ITT population who did not violate any inclusion or exclusion criteria and did not deviate from the protocol in a way that could influence their efficacy assessment. The PP population included 4 of 8 patients in the C3G cohort. Four patients were excluded from the PP analysis in the C3G cohort due to major protocol deviations (less than 75% study drug adherence).

Descriptive statistics are reported as number of patients (*n*) and percentage of total number of patients (%), CFB at week 48, percentage CFB (%CFB) at week 48, and mean (SD). Data are presented for the ITT population, unless otherwise specified. The mean individual CFB was determined by first calculating the CFB in UPCR from baseline to week 48 for each individual patient, and then calculating the mean of these individual results. The %CFB was calculated for each patient as individual CFB divided by baseline UPCR, and then the mean of these individual %CFB was calculated. An alternative approach to calculate %CFB in mean UPCR, which is generally known as a ratio estimator, was also calculated as a mean of change divided by mean of baseline for UPCR.^22^ As a primary sensitivity analysis for the primary end point, individual 24-hour urine total protein (rather than the UPCR) CFB was calculated.

A responder analysis summarized patients with UPCR <500 mg/g (0.5 mg/mg) by each visit and UPCR reduction from baseline by 30% and 50%. The mean of individual %CFB to week 48 was calculated for secondary efficacy (i.e., serum albumin, eGFR, C3, C5a, AH50, and CH50) and pharmacodynamic end points (i.e., C3, C4, C5a, and sC5b-9).

Safety and tolerability of pegcetacoplan were assessed in all patients who received ≥1 dose of pegcetacoplan by monitoring TEAEs throughout the study. Immunogenicity was assessed by antidrug antibody response against the peptide moiety of pegcetacoplan (anti-pegcetacoplan peptide).

## Results

### Patient Population

Demographic and disease characteristics for the C3G cohort (*n =* 8) are shown in Table 1. (IgAN, LN, and PMN cohorts are shown in Supplementary Table S2.) Mean (SD) time since diagnosis was 8.6 (7.6) years. Mean (SD) baseline UPCR was 3.3 (1.7) mg/mg.

**Table 1:** Baseline demographics and disease characteristics of patients with C3G (*N* = 8)^a^

Demographic Characteristics	
Sex, *n* (%)	
Male	3 (37.5)
Female	5 (62.5)
Age, mean (SD), yr	22.5 (8.7)
Weight, mean (SD), kg	76.6 (15.6)
Body mass index, *n =* 7; mean (SD), kg/m^2^	24.9 (5.9)
Race, *n* (%)	
Black/African American	1 (12.5)
White	6 (75.0)
Other^b^	1 (12.5)
Disease characteristics, mean (SD)	
Time since diagnosis, yr	8.6 (7.6)
UPCR from 24-hour urine, mg/mg	3.3 (1.7)
Mean of the 3 spot UPCR, *n =* 6; mg/mg	1.8 (0.8)
Total protein from 24-hour urine, mg/d	5026.5 (2717.1)
eGFR, ml/min per 1.73 m^2^^c^	102.4 (45.5)
Serum albumin, g/dl^d^	3.3 (0.7)
Serum creatinine, mg/dl^e^	1.2 (1.0)
Systolic blood pressure, mm Hg	127.6 (13.2)
Diastolic blood pressure, mm Hg	77.4 (8.8)
Serum C3, mg/dl^f^	44.0 (42.3)
Serum C4, mg/dl^g^	18.5 (7.3)
Plasma sC5b-9, *n =* 7; μg/l^h^	1337.1 (1382.7)
C3 nephritic factor, *n* (%)^i^	
Not detected	7 (87.5)
Detected	1 (12.5)
Concomitant medications, *n* (%)	
RAS blockers	8 (100)
Systemic corticosteroids	3 (37.5)
Immunosuppressants^j^	5 (62.5)
Mycophenolate mofetil	5 (62.5)
Cyclosporin	0
Belimumab	0
Tacrolimus	0
Antibacterials	7 (87.5)
Lipid-modifying agents	4 (50)
Antithrombic/antiplatelet	0
Prior medications, *n* (%)	
RAS blockers	8 (100)
Systemic corticosteroids	4 (50)
Immunosuppressants^j^	5 (62.5)
Mycophenolate mofetil	5 (62.5)
Cyclosporin	0
Belimumab	0
Tacrolimus	0
Antibacterials	3 (37.5)
Lipid-modifying agents	3 (37.5)
Antithrombic/antiplatelet	0

C3G, C3 glomerulopathy; eGFR, estimated glomerular filtration rate; RAS, renin-angiotensin system; sC5b-9, soluble C5b-9; SD, standard deviation; UPCR, urine protein-to-creatinine ratio.

a Number of patients unless mentioned otherwise.

b Other than American Indian or Alaskan Native, Asian, Native Hawaiian, or other Pacific islander.

c eGFR reference value: ≥60 ml/min per 1.73 m^2^.

d Serum albumin reference range: 3.5–5.5 g/dl.

e Serum creatinine reference range: 0.74–1.35 mg/dl for adult men and 0.59–1.04 mg/dl for adult women.

f C3 reference range: 90–180 mg/dl.

g C4 reference range:10–40 mg/dl.

h sC5b-9 normal range: 72–244 μg/l.

i Patients with C3 nephritic factor (ratio of C3 fragment to intact C3) >0.33 were considered to have a detectable value.

j Does not include corticosteroids.

Mean baseline eGFR was ≥60 ml/min per 1.73 m^2^ (102.4 ml/min per 1.73 m^2^) in the C3G cohort. Mean baseline serum C3 (44.0 mg/dl) was markedly low (normal range, 90–180 mg/dl), and mean baseline sC5b-9 levels (1337.1 μg/l) were high (normal range, 72–244 μg/l). C3 nephritic factor was detected in 1 patient.^23^ Baseline biomarkers for other cohorts, including disease-specific biomarkers, are reported in Supplementary Table S2. Most patients reported both prior and concomitant use of renin-angiotensin system blockade (Table 1 and Supplementary Table S2).

### Primary Efficacy

**Figure 3:**
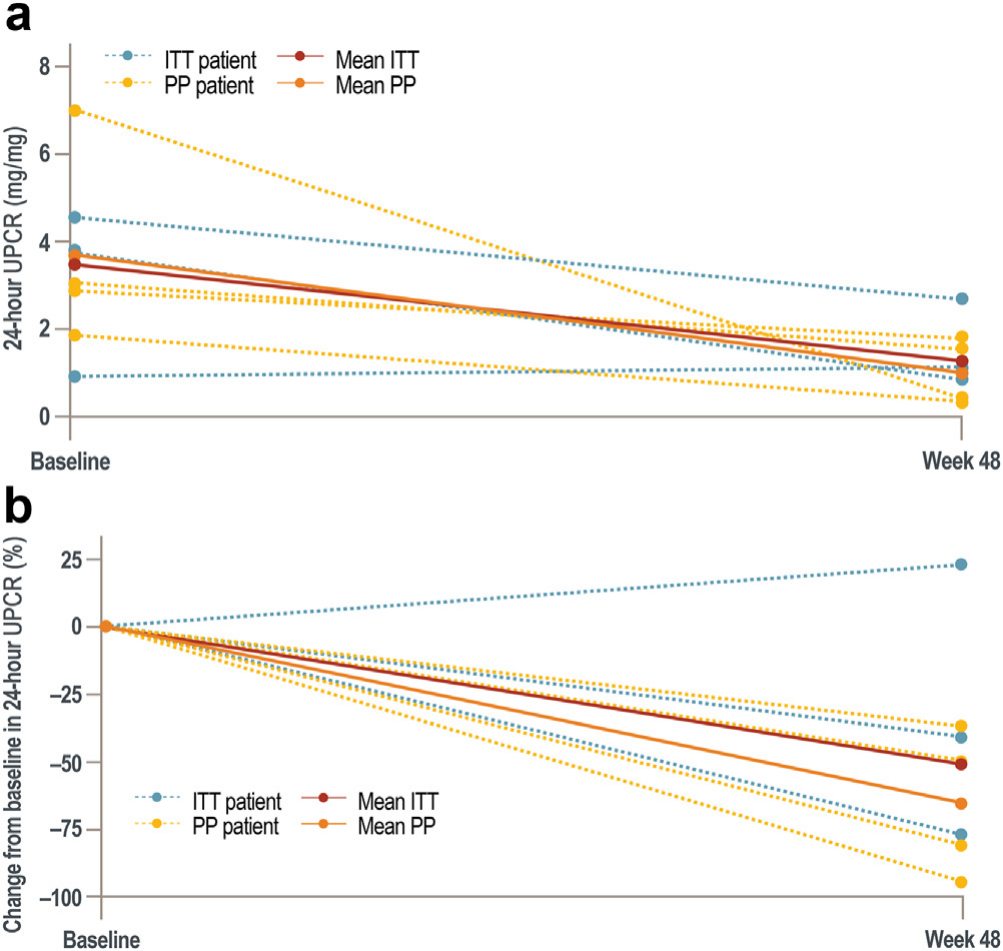
(a) Mean and individual 24-hour UPCR at baseline and week 48 in the C3G cohort (b) Mean and individual percent change from baseline to week 48 in 24-hour UPCR in the C3G cohort. Patients in the C3G cohort of the ITT population with a post-baseline measurement were included in the plot. The PP population includes *n =* 4; those included in ITT only are shown in blue, and those included in both the ITT and PP population are shown in yellow. (a) C3G cohort mean and individual 24-hour UPCR at baseline and week 48. (b) C3G cohort %CFB at week 48 of individual patients’ 24-hour UPCR for both the ITT and PP sets and for the corresponding individual patient values. Baseline ITT (*n =* 8) mean 24-hour UPCR includes 1 patient who did not have follow-up at week 48. C3G, C3 glomerulopathy; CFB, change from baseline; ITT, intent-to-treat; PP, per-protocol; UPCR, urine protein-to-creatinine ratio.

Primary efficacy results for the C3G cohort are shown in Table 2 (IgAN, LN, and PMN cohorts are shown in Supplementary Table S3). In the C3G cohort, UPCR in individual 24-hour urine decreased by a mean (SD) of 2.0 (2.0) mg/mg from baseline to week 48. At week 48, mean (SD) %CFB in individual 24-hour UPCR decreased by 50.9% (39.1) in the ITT population and by 65.4% (26.4) in the PP population (Table 2). This result was consistent with the %CFB in mean UPCR calculated by the ratio estimator (Supplementary Table S4). Individual %CFB in 24-hour UPCR in the C3G cohort ranged from −94.1% to 23.1% at week 48 (Figure 3). In the primary sensitivity analysis, total urine protein at week 48 decreased by 48.3% compared to baseline in the C3G cohort (Supplementary Table S5).

**Table 2:** Primary end points at baseline and week 48 for patients with C3G (ITT and PP populations)

Parameter, mean (SD)	ITT	PP
Baseline^a^		
Number of patients	8	4
24-hour UPCR, mg/mg	3.3 (1.7)	3.5 (2.1)
Week 48		
Number of patients	7	4
24-hour UPCR, mg/mg	1.2 (0.8)	1.0 (0.7)
Individual CFB (SD) in 24-hour UPCR, mg/mg^b^	−2.0 (2.0)	−2.5 (2.5)
Individual %CFB (SD) in 24-hour UPCR^b^^,^^c^	−50.9 (39.1)	−65.4 (26.4)

C3G, complement 3 glomerulopathy; CFB, change from baseline; ITT, intent-to-treat; PP, per-protocol; UPCR, urine protein-to-creatinine ratio.

a Baseline was the most recent result prior to the first dose.

b The means were calculated at each visit with only non-missing values.

c The %CFB was determined for each individual patient as individual CFB divided by baseline UPCR, and then the mean of these individual %CFB was calculated.

### Secondary Efficacy

No patients achieved the predefined complete remission criteria (UPCR <0.2 mg/mg). In the ITT responder analysis (*n =* 8), 6 (75%) patients achieved at least a 30% reduction in UPCR, and 3 (37.5%) patients achieved a partial response (defined as ≥50% reduction in UPCR at week 48). In addition, 2 of 8 (25%) patients had a UPCR <0.5 mg/mg at week 48 (Table 3).

**Table 3:** Clinical responder and UPCR reduction from baseline by 30% and 50%

Clinical responder (UPCR <0.5 mg/mg), *n* (%)		
Analysis visit	UPCR, mg/mg	C3G (*n =* 8)
Baseline	≥0.5	8 (100)
Week 48	<0.5	2 (25.0)
	≥0.5	5 (62.5)

UPCR reduction from baseline by 30% and 50%, *n* (%)		
Analysis visit	UPCR reduction from baseline	C3G (*n =* 8)
Week 48	<30%	1 (12.5)
	≥30%	6 (75.0)
	<50%	4 (50.0)
	≥50%	3 (37.5)

C3G, C3 glomerulopathy; UPCR, urine protein-to-creatinine ratio.

Sample size *n =* 8 at baseline and *n =* 7 at week 48 due to 1 participant who stopped study drug at week 24.

**Figure 4:**
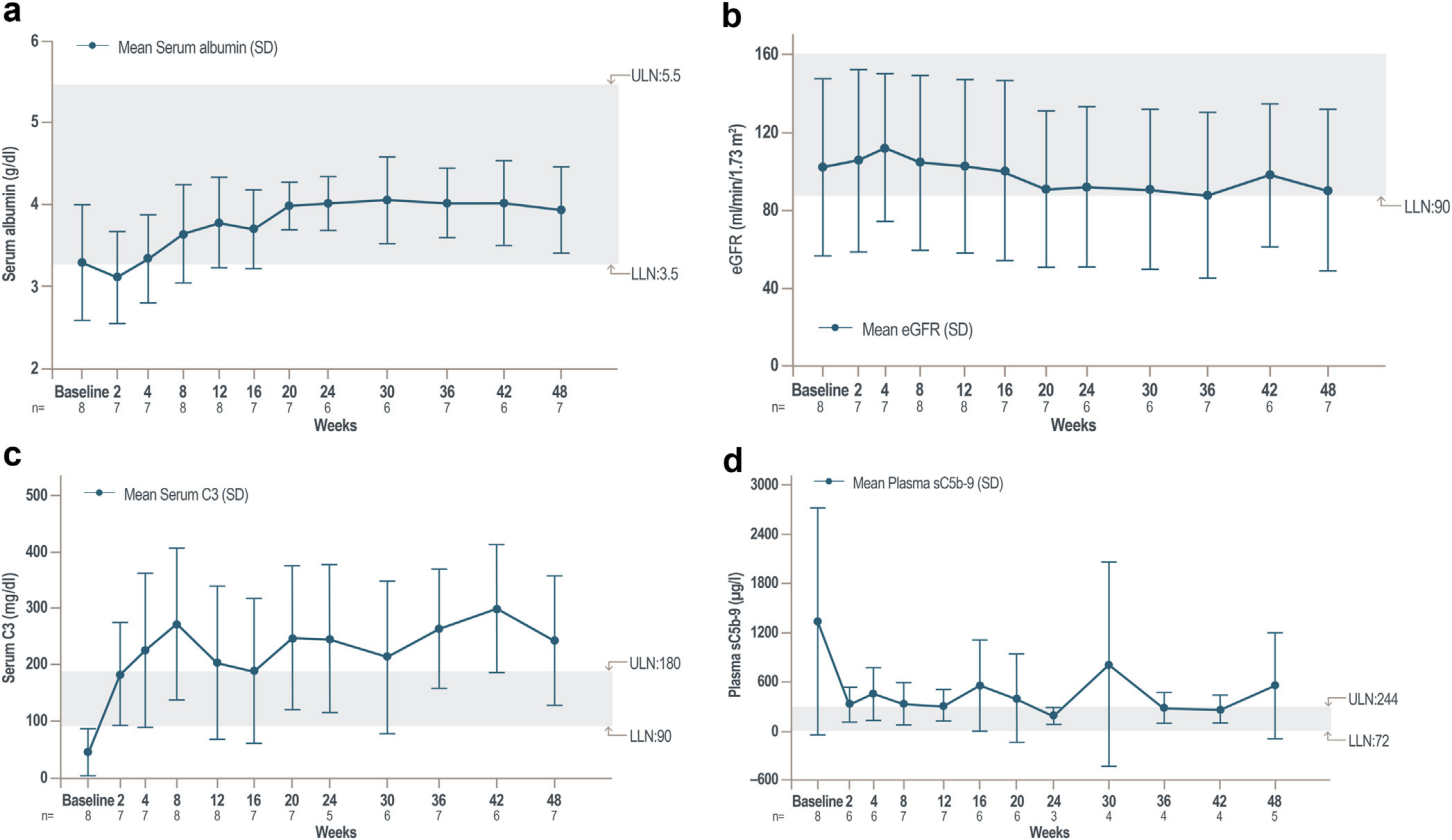
Mean (a) serum albumin, (b) eGFR, (c) serum C3, and (d) plasma sC5b-9 through week 48 in the C3G cohort. Mean (SD) values of (a) serum albumin, (b) eGFR, (c) serum C3, and (d) plasma sC5b-9 levels over time in the C3G cohort (ITT set, *n =* 8). Blue boxes indicate normal reference ranges for variables. C3G, C3 glomerulopathy; eGFR, estimated glomerular filtration rate; ITT, intent-to-treat; LLN, lower limit of normal; sC5b-9, soluble C5b-9; ULN, upper limit of normal.

Secondary efficacy and pharmacodynamic results for the C3G cohort are shown in Table 4 (IgAN, LN, and PMN cohorts are shown in Supplementary Table S6). Mean serum albumin improved at week 48, increasing from 3.3 g/dl (below lower limit of normal) to 3.9 g/dl, which is within normal range (3.5–5.5 g/dl; Table 4).^24^ Improvements in serum albumin were observed as early as week 8 (Figure 4a) and continued through week 48. Mean eGFR was stable throughout the study (Figure 4b). At week 48, 6 (75.0%) patients had stable or improved eGFR (i.e., a change of ≤25%) (Table 4); 1 patient experienced a >25% decline in eGFR, but the eGFR remained within the normal range.^25^ Mean (SD) serum C3 levels increased approximately 6-fold (Table 4), from 44.0 (42.3) mg/dl at baseline to 243.1 (115.2) mg/dl at week 48 (normal C3 range, 90–180 mg/dl) (Figure 4c, Table 4).^26^ The mean (SD) %CFB of sC5b-9 was −57.3% (19.3) at week 48, decreasing from a mean of 1337.1 at baseline to 553.0 μg/l, but remained above the normal range (72–244 μg/l; Table 4). Reductions were observed as early as 2 weeks from treatment initiation (Figure 4d).

**Table 4:** Secondary efficacy and pharmacodynamic end points in the C3G cohort at week 48 (ITT population; *n =* 8)

Parameter, mean (SD)^a^	Baseline^d^	Week 48^d^	%CFB^e^
Serum albumin, *n*; g/dl	8; 3.3 (0.7)	7; 3.9 (0.5)	7; 23.5 (36.7)
Stabilized or improved eGFR^b^, *n*; ≤25% decrease	(–)	6; (75.0)	(–)
Serum C3, *n*; mg/dl	8; 44.0 (42.3)	7; 243.1 (115.2)	7; 666.4 (477.6)
Serum C4, *n*; mg/dl	8; 18.5 (7.3)	6; 18.0 (4.1)	6; 22.2 (78.8)
Plasma C5a, *n*; μg/l	7; 7.5 (3.7)	5; 6.4 (1.7)	5; −18.7 (27.2)
Plasma sC5b-9, *n*; μg/l	7; 1337.1 (1382.7)	5; 553.0 (645.5)	5; −57.3 (19.3)
Serum AH50^c^, *n*; units/ml	8; 38.8 (53.9)	6; 55.7 (42.8)	3; −42.7 (49.8)
Serum CH50, *n*; units/ml	8; 128.6 (121.9)	6; 234.0 (37.6)	6; 1216.8 (2557.8)

AH50, alternative complement pathway hemolytic activity; C3G, complement 3 glomerulopathy; CFB, change from baseline; CH50, classical pathway hemolytic activity, eGFR, estimated glomerular filtration rate; ITT, intent-to-treat; sC5b-9, soluble C5b-9; SD, standard deviation.

a Unless specified otherwise.

b The eGFR was calculated using the Chronic Kidney Disease Epidemiology Collaboration Creatinine Equation.

c Three patients with an AH50 value at baseline of 0 were excluded from the %CFB analysis.

d Reference values: albumin, 3.5–5.5 g/dl; eGFR, ≥60 ml/min per 1.73 m^2^; C3, 90–180 mg/dl; C4, 10–40 mg/dl; C5b-9, 72–244 μg/l; AH50, 77–159 U/ml; CH50, 176–382 U/ml.

e %CFB is the mean of individual percent changes from baseline due to missing data at week 48 (*n =* 8 at baseline and *n =* 7 at week 48 due to one participant who stopped study drug at Week 24).

### Safety (All Cohorts)

Across all cohorts (C3G, IgAN, LN, and PMN), TEAEs were reported in 19 of 21 (90.5%) patients (Tables 5 and 6, Supplementary Tables S7 and S8). None of the TEAEs led to study drug discontinuation or withdrawal, and no deaths occurred during the study. Most TEAEs were deemed mild (38.1%) or moderate (33.3%) by the investigator; no severe TEAEs or serious AEs occurred in the C3G cohort. No serious AEs in any cohort were considered related to pegcetacoplan by the investigator. The most common TEAEs across all cohorts were upper respiratory tract infection (5 patients, 23.8%); injection site erythema, nausea, and headache (4 patients each, 19.0%); and nasopharyngitis, sinusitis, injection site pruritus, diarrhea, vomiting, dizziness, migraine, and acute kidney injury (3 patients each, 14.3%). Across all cohorts, there were no serious AEs or TEAEs of sepsis or meningitis. None of the 21 patients enrolled in the study had positive treatment-emergent or treatment-boosted responses for anti-pegcetacoplan peptide antibodies.

**Table 5:** Summary of TEAEs for patients with C3G and overall study population

Preferred term	C3G (*n =* 8)	Overall (*N* = 21)
Any TEAE, *n* (%)	8 (100)	19 (90.5)
Any serious TEAE, *n* (%)	0	4 (19.0)
Maximum severity of TEAEs, *n* (%)		
Mild	4 (50.0)	8 (38.1)
Moderate	4 (50.0)	7 (33.3)
Severe	0	4 (19.0)
TEAE leading to pegcetacoplan discontinuation	0	0

C3G, C3 glomerulopathy; TEAE, treatment-emergent adverse event.

**Table 6:** TEAEs by preferred term in ≥5% of evaluable patients with C3G and in the overall study population

Preferred term	C3G (*n =* 8)	Overall (*N* = 21)
Any TEAE, *n* (%)	8 (100)	19 (90.5)
Upper respiratory tract infection	2 (25.0)	5 (23.8)
Nasopharyngitis	1 (12.5)	3 (14.3)
Sinusitis	3 (37.5)	3 (14.3)
Pneumonia	1 (12.5)	2 (9.5)
Injection site erythema	1 (12.5)	4 (19.0)
Injection site pruritus	2 (25.0)	3 (14.3)
Fatigue	2 (25.0)	2 (9.5)
Injection site discomfort	0	2 (9.5)
Injection site induration	2 (25.0)	2 (9.5)
Injection site rash	2 (25.0)	2 (9.5)
Pyrexia	2 (25.0)	2 (9.5)
Nausea	3 (37.5)	4 (19.0)
Diarrhea	2 (25.0)	3 (14.3)
Vomiting	3 (37.5)	3 (14.3)
Headache	3 (37.5)	4 (19.0)
Dizziness	1 (12.5)	3 (14.3)
Migraine	2 (25.0)	3 (14.3)
Dyspnea	2 (25.0)	2 (9.5)
Oropharyngeal pain	1 (12.5)	2 (9.5)
Anemia of chronic disease	1 (12.5)	2 (9.5)
Acute kidney injury	0	3 (14.3)
Depression	1 (12.5)	2 (9.5)

C3G, C3 glomerulopathy; TEAE, treatment-emergent adverse event.

## Discussion

The purpose of this study was to explore the preliminary efficacy and safety of pegcetacoplan in patients with C3G, IgAN, LN, and PMN, with efficacy results from the C3G cohort reported herein. As an inhibitor of C3 and C3b, pegcetacoplan targets the primary pathogenic driver of C3G and the complement-mediated disease pathophysiology in IgAN, LN, and PMN. It was hypothesized that pegcetacoplan, by reducing C3 and C3b activation, would reduce proteinuria, particularly in C3G, where the primary driver of disease is dysregulated activation of complement.

In the C3G cohort, pegcetacoplan met the primary efficacy end point of proteinuria (UPCR) reduction from baseline to week 48 within the limitations of the sample size in this initial study. Reduction in proteinuria is an established goal of treatment in glomerulonephritis, with demonstrated correlation to improved kidney survival in chronic kidney disease.^27^ This relationship between proteinuria, measured as total urine protein, and clinical outcomes was demonstrated in a study of 111 patients with C3G conducted by the Spanish Group for the Study of Glomerular Diseases, which observed that a doubling of proteinuria was associated with an increased risk of kidney failure.^28^ This study also demonstrated that a proteinuria reduction of at least 50% was associated with a significantly lower risk of kidney failure.^28^ Across glomerular diseases, studies have demonstrated that a reduction as low as 30% in proteinuria correlates with a reduced risk of end-stage kidney disease.^29^ In the present study, pegcetacoplan resulted in at least a 30% reduction in proteinuria (UPCR) in 6 of 8 (75%) patients with C3G at week 48. Moreover, the mean reduction in proteinuria was 50.9% in the ITT population, which included patients with periods of drug interruption or nonadherence; and 65.4% in the PP population, which included patients with no substantial deviations in dosing. Further, improvement in proteinuria correlated with improvement in serum albumin levels and stable eGFR. It is also notable that 2 patients achieved proteinuria (UPCR) reduction to <0.5 mg/mg, which is often considered a key criterion for complete remission of C3G in the literature.^30^^,^^31^ Complete clinical remission may have been limited in this cohort by the long duration of C3G prior to treatment (mean of 8.6 years), because patients may have had some proteinuria from glomerulosclerosis that is not amenable to immunologic intervention.

Pegcetacoplan increased mean serum C3, providing evidence that pegcetacoplan reduced C3 activation in patients with C3G with aberrant complement regulation. Further, mean sC5b-9 decreased (−57%) after pegcetacoplan administration, indicating inhibition of the downstream terminal complement pathway and C5b-9 assembly.^32^^,^^33^ These data provide preliminary evidence that pegcetacoplan administration mitigates the underlying dysregulation of complement in C3G, with an associated reduction in proteinuria.

It is recognized in clinical practice that patients with C3G with low serum C3 often have low serum CH50 and AH50 due to consumption of C3.^34^ A similar trend of low serum C3 and low serum CH50 and AH50 levels at baseline were also observed in this study. Following pegcetacoplan administration in this population, mean CH50 increased in the C3G cohort to within the normal range. This finding was consistent with the observed impact on CH50 levels seen with pegcetacoplan administration in patients with paroxysmal nocturnal hemoglobinuria, where pegcetacoplan efficacy has been established.^20^^,^^35^ Although CH50 is often utilized in clinical practice to assess extent of complement inhibition with use of C5 inhibitors, the utility of CH50 and other complement assays to assess complement inhibition with pegcetacoplan, a C3 inhibitor, has not been established and requires further exploration.^36^, ^37^, ^38^ Overall, in this study, the findings of proteinuria reduction alongside increased serum C3 and reduced sC5b-9 provide evidence for clinically meaningful complement blockade in patients with C3G who are treated with pegcetacoplan.

Nontargeted immunosuppressant medications (e.g., corticosteroids, calcineurin inhibitors, mycophenolate mofetil) are widely used in C3G in an attempt to control inflammation, despite limited evidence supporting their efficacy in preventing disease progression.^39^ Further, these broad immunosuppressive agents confer an increased risk of infections.^39^ Although many patients in this study received immunosuppressant medications, such as corticosteroids or mycophenolate mofetil, concomitantly with pegcetacoplan, there were no serious AEs of infection, including sepsis or meningitis, over the course of the 48-week study. Overall, pegcetacoplan was found to be generally well-tolerated across all cohorts.

This study has several limitations. First, the small sample size limits the statistical power and may also limit generalizability of the results. Second, patients enrolled in this study were required to have stable or worsening kidney disease, as evidenced by the required proteinuria threshold for the study (>750 mg/g 24-hour UPCR), despite being on stable and optimized treatment for at least 2 months prior to initiating pegcetacoplan. As part of this treatment, most patients received concomitant immunosuppressants (i.e., corticosteroids or mycophenolate mofetil), which can lead to improved kidney outcomes for patients with C3G.^31^ However, such improvements were not seen in this patient population with a stable and optimized regimen prior to inclusion into this study as demonstrated by their nephrotic-range proteinuria at baseline (mean [SD] 24-hour UPCR of 3.3 [1.7]).

Pegcetacoplan reduced proteinuria in patients with C3G while maintaining stable kidney function over 48 weeks of treatment. These results support further clinical development of pegcetacoplan for the treatment of C3G, as well as other conditions with overlapping pathogenesis, such as primary or idiopathic immune complex membranoproliferative glomerulonephritis. Of note, primary idiopathic immune complex membranoproliferative glomerulonephritis, with the presence of C3 and immunoglobulin, has similar clinical manifestations to C3G, with biopsy evidence that some patients transition between diagnoses.^40^ Further clinical development of pegcetacoplan is ongoing and will aim to target a broad spectrum of patients with C3G and primary idiopathic immune complex membranoproliferative glomerulonephritis, including pediatric patients and those with post-transplant recurrence.

## Disclosure

BPD has served as a consultant for Apellis Pharmaceuticals, Inc., and Alexion Pharmaceuticals. LAG received research support from Alexion Pharmaceuticals, Advicenne, AbbVie, Apellis Pharmaceuticals, Inc., Aurinia Pharmaceuticals, Inc., Reata Pharmaceuticals, Vertex Pharmaceuticals, and Roche; and was a consultant for Caratherapeutics, Novartis, Advicenne, Alexion Pharmaceuticals, Roche, Aurinia Pharmaceuticals, Inc., Otsuka Pharmaceutical Co., Ltd., and Arrowhead Pharmaceuticals. SR has received research support from Uniqure; was a consultant for Apellis Pharmaceuticals, Inc.; was an advisory board member for Alexion Pharmaceuticals, Agios Pharmaceuticals, Apellis Pharmaceuticals, Inc., Biomarin Pharmaceutical, Inc., Novo Nordisk, and Sanofi Genentech; and was a speakers bureau member for Alexion Pharmaceuticals, Agios Pharmaceuticals, Novo Nordisk, and Sanofi Genentech. CK, LL, and YZ are employees of and hold stock in Apellis Pharmaceuticals, Inc. LH has no disclosures to report.

## Supplementary Material

Supplementary File (PDF)

**Table S1.** Key exclusion criteria and inclusion criteria for patients with other complement-mediated diseases.

**Table S2.** Baseline demographics and disease characteristics of patients with complement-mediated diseases.

**Table S3.** Primary end points at baseline and week 48 (ITT and PP populations) for patients with other complement-mediated diseases.

**Table S4.** Primary end points at baseline and week 48 (ITT and PP populations), %CFB in mean UPCRa.

**Table S5.** Primary sensitivity analysis using total urinary protein from 24-hour urine samples at baseline and week 48 (ITT population).

**Table S6.** Secondary efficacy and pharmacodynamic end points in the IgAN, LN, and PMN cohorts at week 48 (ITT population).

**Table S7.** Summary of treatment-emergent adverse events in patients with other complement-mediated diseases.

**Table S8.** Treatment-emergent adverse events by preferred term in ≥5% of evaluable patients with other complement-mediated diseases.
