# Supplementary material for: Clinical Safety and Efficacy of Pegcetacoplan in a Phase 2 Study of Patients with C3 Glomerulopathy and Other Complement-Mediated Glomerular Diseases
Source: Kidney Int Rep. 2023 Aug 25;8(11):2284–93. doi: 10.1016/j.ekir.2023.08.033 (PMC10658235; doi:10.1016/j.ekir.2023.08.033)
Supplement: Supplementary File (PDF) [file mmc1.pdf]

SUPPLEMENTAL MATERIAL

Supplemental Table S1. Key exclusion criteria and inclusion criteria for patients with other complement-mediated diseases

Inclusion Criteria for Patients with IgAN, LN, and PMN

- Age ≥18 years
- Diagnosis confirmed by kidney biopsy and required measurements performed prior to study participation
    - IgAN: Prior biopsy results for C3 and C4d staining
    - LN: Diagnostic biopsy showing proliferative focal, diffuse, or membranous lesions (Class III, IV, or V, respectively) by kidney biopsy. Patient needed either a biopsy in the last 6 months or evidence of disease activity (nephritic changes on urinalysis or nephrotic changes).
    - PMN: PLA2R-positive titer plus nephrotic-range proteinuria (defined as UPCR >2350 mg/g)
- Proteinuria >750 mg/g UPCR (on 24-hour urine collection) collected at the first screening
- eGFR ≥30 mL/min/1.73 m^2

Exclusion Criteria for All Cohorts

- Absolute neutrophil count <1000 cells/mm^3
- ALT or AST >3.0 times the ULN
- Previous exposure to pegcetacoplan
- Diagnosis of malignancy (except for cured basal or squamous cell skin cancer, curatively treated in situ disease, or 5 or more years disease free from cancer and not receiving ongoing treatment)
- Diagnosis of HIV, active HBV or HCV infection or positive serology
- History of solid organ transplant
- Kidney disease secondary to another condition (e.g., infection, malignancy, monoclonal gammopathy, or a medication)

- Participation in any other investigational drug trial or exposure to other investigational agent, device, or procedure within 30 days prior to screening period
- Pregnant, breastfeeding, or intention to conceive during the study

ALT, alanine aminotransferase; AST, aspartate aminotransferase; eGFR, estimated glomerular filtration rate; HBV, hepatitis B virus; HCV, hepatitis C virus; HIV, human immunodeficiency virus; IgAN, immunoglobulin A nephropathy; LN, lupus nephritis; PLA2R, phospholipase A2 receptor; PMN, primary membranous nephropathy; ULN, upper limit of normal; UPCR, urine protein-to-creatinine ratio.

Supplemental Table S2. Baseline demographics and disease characteristics of patients with complement-mediated diseases

	IgAN (*n* = 6)[a]	LN (*n* = 2)[a]	PMN (*n* = 5)[a]
**Demographic characteristics**			
**Sex, *n* (%)**			
Male	5 (83.3)	2 (100.0)	4 (80.0)
Female	1 (16.7)	0	1 (20.0)
**Age, mean (SD), y**	45.2 (14.2)	44.5 (10.6)	62.2 (7.4)
**Weight, mean (SD), kg**	92.6 (20.7)	119.7 (47.2)	109.1 (11.9)
**Body mass index, *n*; mean (SD), kg/m^2**	4; 27.6 (4.3)	1; 29.2 (–)	4; 34.6 (2.3)
**Race, *n* (%)**			
Black/African American	1 (16.7)	1 (50.0)	1 (20.0)
White	4 (66.7)	0	4 (80.0)
Other[b]	1 (16.7)	1 (50.0)	0
**Disease characteristics, mean (SD)**			
Time since diagnosis, y	4.7 (4.6)	5.7 (1.8)	6.1 (7.5)
UPCR from 24-hour urine, mg/mg	1.3 (0.5)	4.9 (5.3)	6.4 (4.5)
Mean of the three spot UPCR, *n*; mg/mg	4; 1.0 (0.4)	1; 6.2 (–)	4; 8.2 (5.1)
Total protein from 24-hour urine, mg/d	2974.2 (1235.0)	11,293.5 (11,587.4)	10,577.4 (7226.1)
eGFR, mL/min/1.73 m^2[c]	67.7 (31.0)	55.6 (35.8)	54.9 (16.7)
Serum albumin, g/dL[d]	4.2 (0.6)	2.8 (0.4)	2.8 (1.0)
Serum creatinine, mg/dL[e]	1.4 (0.5)	1.9 (1.0)	1.4 (0.4)
Systolic blood pressure, mmHg	119.8 (10.9)	130.0 (7.1)	135.8 (10.0)
Diastolic blood pressure, mmHg	73.8 (7.9)	83.5 (2.1)	79.2 (11.7)
Serum C3, mg/dL[f]	139.7 (35.8)	105.5 (0.7)	127.8 (23.0)

Serum C4, *n*; mg/dL[g]	3, 23.0 (2.7)	1, 22.0 (–)	5, 28.4 (5.2)
Plasma sC5b-9, *n*; µg/L[h]	6; 160.8 (33.0)	2; 400.5 (204.4)	5; 193.8 (114.2)
**C3 nephritic factor, *n* (%)[i]**			
Not detected	NR	NR	NR
Detected	NR	NR	NR
**Concomitant medications, *n* (%)**			
RAS blockers	6 (100)	1 (50)	4 (80)
Systemic corticosteroids	1 (16.7)	0	5 (100)
Immunosuppressants[j]	0	1 (50)	2 (40)
Mycophenolate mofetil	0	0	0
Cyclosporin	0	0	2 (40)
Belimumab	0	0	0
Tacrolimus	0	1 (50)	0
Antibacterials	2 (33.3)	1 (50)	4 (80)
Lipid-modifying agents	6 (100)	1 (50)	3 (60)
Antithrombic/antiplatelet	1 (16.7)	0	4 (80)
**Prior medications, *n* (%)**			
RAS blockers	6 (100)	1 (50)	4 (80)
Systemic corticosteroids	0	0	2 (40)
Immunosuppressants[j]	0	2 (100)	2 (40)
Mycophenolate mofetil	0	1 (50)	0
Cyclosporin	0	0	2 (40)
Belimumab	0	1 (50)	0
Tacrolimus	0	1 (50)	0
Antibacterials	0	0	0
Lipid-modifying agents	6 (100)	1 (50)	3 (60)

Antithrombic/antiplatelet	1 (16.7)	0	3 (60)

eGFR, estimated glomerular filtration rate; IgAN, immunoglobulin A nephropathy; LN, lupus nephritis; NR, not reported; PMN, primary membranous nephropathy; RAS, renin-angiotensin system, sC5b-9, soluble C5b-9; SD, standard deviation; UPCR, urine protein-to-creatinine ratio.

[a]Number of patients unless mentioned otherwise. [b]Other than American Indian or Alaskan Native, Asian, Native Hawaiian or other Pacific islander. [c]eGFR reference value: ≥60 mL/min/1.73 m^2. [d]Serum albumin reference range: 3.5–5.5 g/dL. [e]Serum creatinine reference range: 0.74–1.35 mg/dL for adult men and 0.59–1.04 mg/dL for adult women. [f]C3 reference range: 90–180 mg/dL. [g]C4 reference range: 10–40 mg/dL. [h]sC5b-9 normal range: 72–244 μg/L. [i]Patients with C3 nephritic factor (ratio of C3 fragment to intact C3) >0.33 were considered to have a detectable value. [j]Does not include corticosteroids.

Supplemental Table S3. Primary endpoints at baseline and Week 48 (ITT and PP populations) for patients with other complement-mediated diseases

	ITT		
**Parameter, mean (SD)**	**IgAN**	**LN**	**PMN**
**Baseline[a]**			
Number of patients	6	2	5
24-hour UPCR, mg/mg	1.3 (0.5)	4.9 (5.3)	6.4 (4.5)
**Week 48**			
Number of patients	5	1	1
24-hour UPCR, mg/mg	1.2 (0.5)	9.0	1.5
Individual CFB (SD) in 24-hour UPCR, mg/mg[b]	–0.1 (0.8)	0.4 (–)[d]	0.3 (–)[d]
Individual %CFB (SD) in 24-hour UPCR[b,c]	–1.1 (50.3)	(–)[d]	(–)[d]
	**PP**		
**Parameter, mean (SD)**	**IgAN**	**LN**	**PMN**
**Baseline[a]**			
Number of patients	6	2	4
24-hour UPCR, mg/mg	1.3 (0.5)	4.9 (5.3)	7.7 (4.0)
**Week 48**			

Number of patients	5	1	0
24-hour UPCR, mg/mg	1.2 (0.5)	(–)[d]	(–)[d]
Individual CFB (SD) in 24-hour UPCR, mg/mg[b]	–0.1 (0.8)	0.4 (–)[d]	(–)[d]
Individual %CFB (SD) in 24-hour UPCR[b,c]	–1.1 (50.3)	(–)[d]	(–)[d]

CFB, change from baseline; IgAN, immunoglobulin A nephropathy; ITT, intent-to-treat; LN, lupus nephritis; PMN, primary membranous nephropathy; PP, per-protocol; SD, standard deviation; UPCR, urine protein-to-creatinine ratio.

[a]Baseline was the most recent result prior to the first dose. [b]The means were calculated at each visit regardless of whether patients had a non-missing value. [c]The %CFB was determined for each individual patient as individual CFB over baseline UPCR, and then the mean of these individual %CFB was calculated. [d]Data are not available or reported because *n* = 1 or *n* = 0.

Supplemental Table S4. Primary endpoints at baseline and Week 48 (ITT and PP populations), %CFB in mean UPCR[a]

	ITT			
**Parameter, mean (SD)**	**C3G**	**IgAN**	**LN**	**PMN**
**Baseline[b]**				
Number of patients	8	6	2	5
24-hour UPCR, mg/mg	3.3 (1.7)	1.3 (0.5)	4.9 (5.3)	6.4 (4.5)
**Week 48**				
Number of patients	7	5	1	1
24-hour UPCR, mg/mg	1.2 (0.8)	1.2 (0.5)	9.0[d]	1.5[d]
%CFB in mean 24-hour UPCR[c]	–63.8	–5.2	(–)[d]	(–)[d]
	**PP**			
**Parameter, mean (SD)**	**C3G**	**IgAN**	**LN**	**PMN**
**Baseline[b]**				
Number of patients	4	6	2	4
24-hour UPCR, mg/mg	3.5 (2.1)	1.3 (0.5)	4.9 (5.3)	7.7 (4.0)
**Week 48**				
Number of patients	4	5	1	0

24-hour UPCR, mg/mg	1.0 (0.7)	1.2 (0.5)	(–)[d]	(–)[d]
%CFB in mean 24-hour UPCR[c]	–72.2	–5.2	(–)[d]	(–)[d]

C3G, complement 3 glomerulopathy; CFB, change from baseline; IgAN, immunoglobulin A nephropathy; ITT, intent-to-treat; LN, lupus nephritis; PMN, primary membranous nephropathy; PP, per-protocol; SD, standard deviation; UPCR, urine protein-to-creatinine ratio.

[a]An alternative approach to calculate %CFB in mean uPCR, which is generally known as a ratio estimator, was also calculated as a mean of change over mean of baseline for UPCR. [b]Baseline was the most recent result prior to the first dose. [c]The means were calculated at each visit regardless of whether patients had a non-missing value. [d]Means or SDs are not available because $n = 1$ or $n = 0$.

Supplemental Table S5. Primary sensitivity analysis using total urinary protein from 24-hour urine samples at baseline and Week 48 (ITT population)

**Parameter, mean (SD)**	**C3G**	**IgAN**	**LN**	**PMN**
**Baseline**[a]				
Number of patients	8	6	2	5
Protein excretion rate, mg/day	5026.5 (2717.1)	2974.2 (1235.0)	11,293.5 (11,587.4)	10,577.4 (7226.1)
**Week 48**				
Number of patients	7	5	1	1
Protein excretion rate, mg/day	1698.4 (1095.6)	2676.4 (1408.1)	8208.0[c]	4228.0[c]
Individual CFB	–3731.9 (3251.6)	–673.6 (1160.1)	11,279.0[c]	1593.0[c]
Individual %CFB[b]	–48.3 (68.9)	–20.6 (31.2)	(–)[c]	(–)[c]

C3G, complement 3 glomerulopathy; CFB, change from baseline; IgAN, immunoglobulin A nephropathy; ITT, intent-to-treat; LN: lupus nephritis; PMN, primary membranous nephropathy; SD, standard deviation.

[a]Baseline was the most recent result prior to the first dose. [b]The means were calculated at each visit regardless of whether patients had a non-missing value. [c]Means or SDs are not available because *n* = 1.

Supplemental Table S6. Secondary efficacy and pharmacodynamic endpoints in the IgAN, LN, and PMN cohorts at Week 48 (ITT population)

Parameter, mean (SD)[a]	Baseline[d]			Week 48[d]			%CFB[e]
	IgAN (n = 6)	LN (n = 2)	PMN (n = 5)	IgAN (n = 6)	LN (n = 0)	PMN (n = 2)	IgAN (n = 6)
Serum albumin	4.2 (0.6)	2.8 (0.4)	2.8 (1.0)	4.0 (0.1)	(–)	4.0 (0.1)	–2.4 (13.8)
Stabilized or improved eGFR[b,c]: ≤25% decrease	(–)	(–)	(–)	3 (50.0)	(–)	1 (20.0)	(–)
Serum C3, mg/dL	139.7 (35.8)	105.5 (0.7)	127.8 (23.0)	292.8 (128.3)	(–)	476.0 (0.0)	113.8 (73.1)
Serum C4, *n*, mg/dL	3; 23.0 (2.7)	1; 22.0 (–)	5; 28.4 (5.2)	3; 18.0 (3.5)	(–)	1; 25.0 (–)	3; –22.2 (6.9)

CFB, change from baseline; eGFR: estimated glomerular filtration rate; IgAN, immunoglobulin A nephropathy; ITT, intent-to-treat; LN, lupus nephritis; PMN, primary membranous nephropathy; SD, standard deviation.

[a]Unless specified otherwise. [b]The eGFR was calculated using the CKD-EPI creatinine equation. [c]Number of patients (%). [d]Reference values: albumin, 3.5–5.5 g/dL; eGFR, ≥60 mL/min/1.73m^2; C3, 90–180 mg/dL; C4, 10–40 mg/dL; [e]Data are not available or reported for LN and PMN because n = 1 or n = 0.

Supplemental Table S7. Summary of treatment-emergent adverse events in patients with other complement-mediated diseases

**Preferred term**	**IgAN (*n* = 6)**	**LN (*n* = 2)**	**PMN (*n* = 5)**
**Any TEAE, *n* (%)**	5 (83.3)	1 (50.0)	5 (100)
**Any serious TEAE, *n* (%)**	0	0	4 (80.0)
**Maximum severity of TEAEs, *n* (%)**			
Mild	3 (50.0)	1 (50.0)	0
Moderate	1 (16.7)	0	2 (40.0)
Severe	1 (16.7)	0	3 (60.0)
**TEAE leading to pegcetacoplan discontinuation**	0	0	0

IgAN, immunoglobulin A nephropathy; LN, lupus nephritis; PMN, primary membranous nephropathy; TEAE, treatment-emergent adverse event.

Supplemental Table S8. Treatment-emergent adverse events by preferred term in ≥5% of evaluable patients with other complement-mediated diseases

**Preferred Term**	**IgAN (*n* = 6)**	**LN (*n* = 2)**	**PMN (*n* = 5)**
**Any TEAE, *n* (%)**	5 (83.3)	1 (50.0)	5 (100)
Upper respiratory tract infection	2 (33.3)	0	1 (20.0)
Nasopharyngitis	0	0	2 (40.0)
Sinusitis	0	0	0
Pneumonia	0	0	1 (20.0)
Injection site erythema	2 (33.3)	0	1 (20.0)
Injection site pruritus	0	0	1 (20.0)
Fatigue	0	0	0
Injection site discomfort	2 (33.3)	0	0
Injection site induration	0	0	0
Injection site rash	0	0	0

Pyrexia	0	0	0
Nausea	1 (16.7)	0	0
Diarrhea	0	0	1 (20.0)
Vomiting	0	0	0
Headache	0	1 (50.0)	0
Dizziness	1 (16.7)	0	1 (20.0)
Migraine	1 (16.7)	0	0
Dyspnea	0	0	0
Oropharyngeal pain	1 (16.7)	0	0
Anemia of chronic disease	0	0	1 (20.0)
Acute kidney injury	0	0	3 (60.0)
Depression	1 (16.7)	0	0

IgAN, immunoglobulin A nephropathy; LN, lupus nephritis; PMN, primary membranous nephropathy; TEAE, treatment-emergent adverse event.
